# *CD24*, *CD27*, *CD36* and *CD302* gene expression for outcome prediction in patients with multiple myeloma

**DOI:** 10.18632/oncotarget.22131

**Published:** 2017-10-30

**Authors:** Elina Alaterre, Sebastien Raimbault, Hartmut Goldschmidt, Salahedine Bouhya, Guilhem Requirand, Nicolas Robert, Stéphanie Boireau, Anja Seckinger, Dirk Hose, Bernard Klein, Jérôme Moreaux

**Affiliations:** ^1^ Department of Biological Haematology, CHU Montpellier, Montpellier, France; ^2^ Institute of Human Genetics, CNRS-UM UMR9002, Montpellier, France; ^3^ University of Montpellier, UFR Medecine, Montpellier, France; ^4^ Medizinische Klinik und Poliklinik V, Universitätsklinikum Heidelberg, Heidelberg, Germany; ^5^ Nationales Centrum für Tumorerkrankungen, Heidelberg, Germany; ^6^ HORIBA Medical, Parc Euromédecine, Montpellier, France; ^7^ CHU Montpellier, Department of Clinical Hematology, Montpellier, France

**Keywords:** multiple myeloma, prognostic factor, gene expression profiling, cluster differentiation

## Abstract

Multiple myeloma (MM) is a B cell neoplasia characterized by clonal plasma cell (PC) proliferation. Minimal residual disease monitoring by multi-parameter flow cytometry is a powerful tool for predicting treatment efficacy and MM outcome.

In this study, we compared CD antigens expression between normal and malignant plasma cells to identify new potential markers to discriminate normal from malignant plasma cells, new potential therapeutic targets for monoclonal-based treatments and new prognostic factors. Nine genes were significantly overexpressed and 16 were significantly downregulated in MMC compared with BMPC (ratio ≥2; FDR *CD24*, *CD27*, *CD36* and *CD302*) was associated with a prognostic value in two independent cohorts of patients with MM (HM cohort and TT2 cohort, n=345). The expression level of these four genes was then used to develop a CD gene risk score that classified patients in two groups with different survival (*P* = 2.06E-6) in the HM training cohort. The prognostic value of the CD gene risk score was validated in two independent cohorts of patients with MM (TT2 cohort and HOVON65/GMMGHD4 cohort, n=282 patients). The CD gene risk score remained a prognostic factor that separated patients in two groups with significantly different overall survival also when using publicly available data from a cohort of relapsing patients treated with bortezomib (n=188). In conclusion, the CD gene risk score allows identifying high risk patients with MM based on *CD24*, *CD27*, *CD36* and *CD302* expression and could represent a powerful tool for simple outcome prediction in MM.

## INTRODUCTION

Multiple myeloma (MM) is a B cell neoplasia characterized by accumulation of clonal malignant plasma cells (MMC) in bone marrow. In the United States and in Europe, MM affects 20 000 new patients per year [1, 2]. MM is the second most prevalent hematological malignancy and remains an incurable disease in most cases with a median survival of 5-7 years [1]. The treatment of relapsed/refractory patients is challenging. Monoclonal antibodies associated with other agents, such as immunomodulatory drugs or proteasome inhibitors, are showing promising results [3, 4]. However, the long-term clinical outcome of patients with MM is still heterogeneous.

The cluster of differentiation (CD) includes cell surface molecules that are used for cell immunophenotyping. CD19, CD20, CD27, CD28, CD56 and CD117 are usually employed to discriminate normal bone marrow plasma cells (BMPC) from MMC [5]. The expression of these CD markers is not homogeneous in all MMC. Indeed, they are not aberrantly expressed in all patients with MM and their expression level is heterogeneous among patients and MM subclones [6–9]. Higher expression of CD117 and CD56 was identified in patients with hyperdiploidy whereas high CD56 expression and low CD117 expression was associated with t(4;14) MM patients. At the opposite, MAF molecular subgroup was characterized by high CD117 expression and low CD56 expression. CD20 expression is negatively correlated with amplification of chromosome 1q21 [10]. In this study, we analyzed and compared the expression of genes coding for CD antigens in BMPC and primary MMC from newly diagnosed patients using microarrays. We identified new CD genes that are differentially expressed between normal and malignant plasma cells. Furthermore, we found that the expression level of *CD24*, *CD27*, *CD36* and *CD302* has a prognostic value in independent cohorts of newly diagnosed patients with MM. Based on the expression level of *CD24*, *CD27*, *CD36* and *CD302*, we then developed a new CD gene risk score that allows the identification of high-risk patients with MM at diagnosis and at relapse.

## RESULTS

### CD genes differentially expressed in normal BMPC and MMC

**Figure 1:**
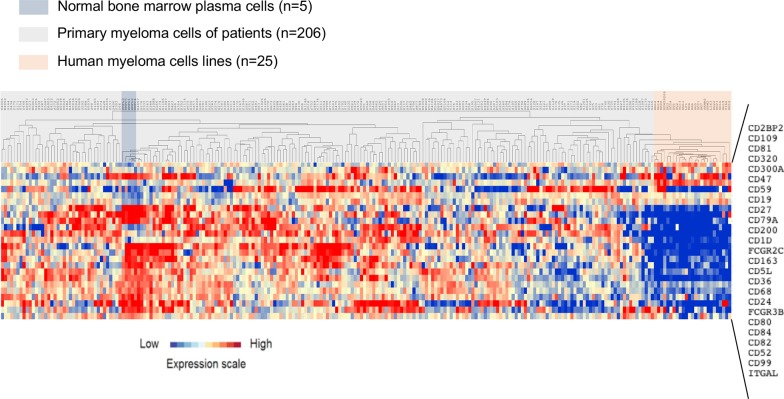
Clustering of CD gene expression in normal BMPC, MMC and HMCL The expression of 25 genes from 266 probesets was significantly deregulated in MMC of patients with MM (HM cohort) at diagnosis compared with normal BMPC and HMLC. The expression level of each gene is color-coded (red, higher and blue, lower expression than the mean; the color intensity represents the magnitude of deviation from the mean). MMC, normal BMPC and HMCL samples are highlighted in gray, blue and red, respectively.

A list of 266 probesets representative of genes encoding CD molecules was defined using the Human Cell Differentiation Molecules database (http://www.hcdm.org) (Supplementary Table 1). Comparison of CD gene expression in BMPC from healthy controls and MMC samples from patients with MM (HM cohort) using Significance Analysis of Microarrays (SAM) showed that nine CD genes were significantly overexpressed in MMC compared with BMPC and 16 were significantly downregulated [ratio ≥2; False Discovery Rate (FDR) <0.05; 1000 permutations] (Tables 1 and 2) (Figure 1). Among the CD genes significantly overexpressed in MMC samples, the biological function of *CD200*, *CD1d*, *CD47*, *CD59* and *CD32* (*Fc* γ*RII*) in MM has been already investigated [11–20]. On the other hand, CD109, CD2BP2, CD300a and CD320 could represent new potential MMC CD markers (Table 1). Affymetrix gene expression of CD109 and CD300a was validated at protein level using panel of 11 HMCL (Supplementary Figure 1). CD300a overexpression in malignant compared to normal PCs was also confirmed by flow cytometry in a newly diagnosed patient (Supplementary Figure 1). The expression of *CD320* and *CD2BP2* was also significantly higher in human MM cell lines (HMCL; n=25) [ratio≥ 2, FDR< 0.05; 1000 permutations] compared with MMC, suggesting that they could be involved in MM progression (Figure 1) (Supplementary Table 2A).

**Table 1:** Cluster differentiation genes significantly overexpressed in MMC compared with normal BMPC

Probeset	Gene	Ratio	FDR	Affymetrix description
229900_at	CD109	2.33	1.34	CD109 molecule
205789_at	CD1d	2.12	1.83	CD1d molecule
209583_s_at	CD200	5.79	0.28	CD200 molecule
202256_at	CD2BP2	2.26	0.00	CD2 (cytoplasmic tail) binding protein 2
217078_s_at	CD300a	5.80	1.08	CD300a molecule
218529_at	CD320	2.00	0.28	CD320 molecule
213857_s_at	CD47	3.70	0.00	CD47 molecule
200985_s_at	CD59	2.85	0.00	CD59 molecule, complement regulatory protein
211395_x_at	FCGR2c	2.62	0.28	Fc fragment of IgG, low affinity IIc, receptor for (CD32) (gene/pseudogene)

Gene expression was profiled in MMC samples from the HM cohort (n=206 patients with MM) and BMPC samples (healthy controls; n=7) using Affymetrix U133 plus 2.0 microarrays. Genes that are differentially expressed between malignant and normal cells were identified using SAM supervised unpaired analysis with a 5% false discovery rate.

**Table 2:** Cluster differentiation genes that are downregulated in MMC compared with normal BMPC

Probeset	Gene	Ratio	FDR	Affymetrix description
215049_x_at	CD163	0.16	0.00	CD163 molecule
206398_s_at	CD19	0.32	0.00	CD19 molecule
216379_x_at	CD24	0.40	0.00	CD24 molecule
228766_at	CD36	0.22	0.00	CD36 molecule (thrombospondin receptor)
34210_at	CD52	0.28	0.00	CD52 molecule
206680_at	CD5L	0.25	0.00	CD5 molecule-like
203507_at	CD68	0.27	0.00	CD68 molecule /// small nucleolar RNA, H/ACA box 67
205049_s_at	CD79a	0.37	0.00	CD79a molecule, immunoglobulin-associated alpha
207176_s_at	CD80	0.47	0.00	CD80 molecule
200675_at	CD81	0.29	0.00	CD81 molecule
203904_x_at	CD82	0.23	0.00	CD82 molecule
205988_at	CD84	0.43	0.00	CD84 molecule
201029_s_at	CD99	0.37	0.00	CD99 molecule
204007_at	FCGR3b	0.42	0.00	Fc fragment of IgG, low affinity IIIb, receptor (CD16b)
213475_s_at	ITGAL	0.47	0.00	integrin, alpha L (antigen CD11A (p180), lymphocyte function-associated antigen 1; alpha polypeptide)
206150_at	TNFRSF7	0.25	0.00	CD27 molecule

Gene expression was profiled in MMC samples from the HM cohort (n=206 patients with MM) and BMPC samples (healthy controls; n=7) using Affymetrix U133 plus 2.0 microarrays. Genes that are significantly differentially expressed were identified using SAM supervised unpaired analysis with a 5% false discovery rate.

Among the 16 CD genes that were significantly downregulated [ratio ≥2; FDR <0.05; 1000 permutations] in MMC compared with normal BMPC samples (Table 2), CD19 and CD27 are already used to discriminate BMPC from MMC [21–23]. Moreover, 11 of these 16 CD genes were also significantly downregulated in HMCL [ratio≥ 2, FDR< 0.05; 1000 permutations] compared with primary MMC (Figure 1) (Supplementary Table 2B).

### Prognostic value of CD gene expression in MM and development of a CD gene risk score

**Figure 2:**
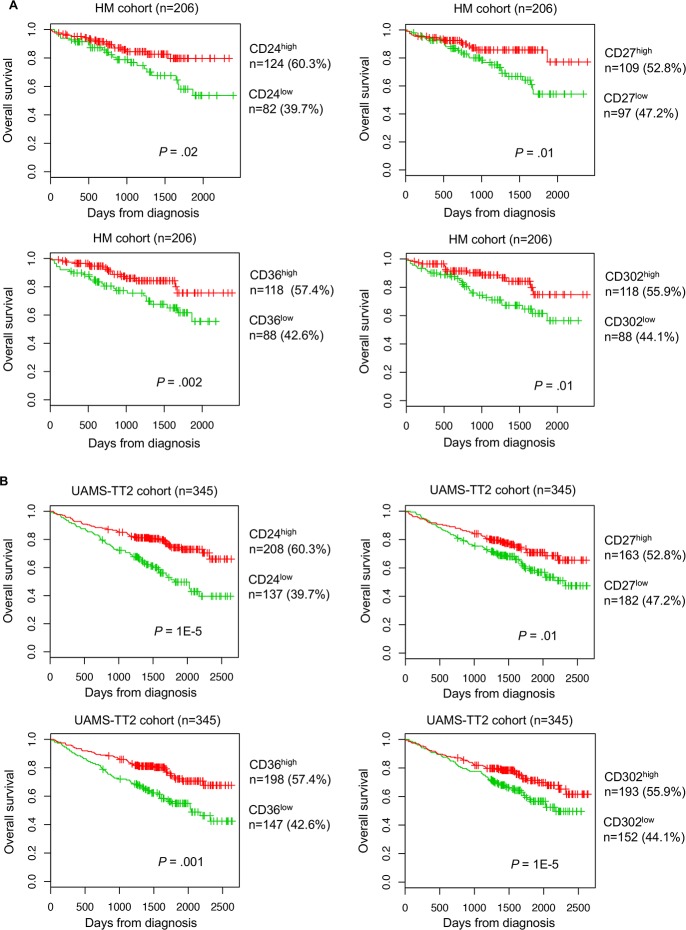
Prognostic value of CD24, CD27, CD36 and CD302 expression level in patients with MM Overall survivalestimations using Kaplan-Meier curves in patients with MM with high (red) or low (green) expression of CD24, CD27, CD36 and CD302 in two independent cohorts: **(A)** HM training cohort and **(B)** TT2 validation cohort. Patients with high expression of CD24, CD27, CD36 and CD302 showed longer overall survival compared with patients with low expression.

Using the Maxstat R function and the Benjamini Hochberg multiple testing correction [24], high expression of four genes (*CD24*, *CD27*, *CD36* and *CD302*), among the 266 CD probesets, was associated with longer overall survival (OS) in both training and validation cohorts (HM cohort: n=206, and TT2 cohort: n=345, respectively) (Figure 2A and 2B).

Moreover, by classifying patients with MM in different molecular subgroups [25], we found that *CD24, CD27* and *CD36* were significantly downregulated in MMC samples from patients with MM classified in the proliferation subgroup. *CD24* was also significantly downregulated in the hyperdiploid subgroup, whereas *CD27* and *CD302* were downregulated in the CD1 subgroup. Conversely, in the CD2 subgroup, *CD27* was significantly overexpressed. Finally, *CD302* was significantly overexpressed in the low bone disease subgroup and downregulated in the MAF and CD1 subgroups (Supplementary Figure 2). We validated the heterogeneous expression of CD24, CD36 and CD302 in MM (Supplementary Figure 8) by real-time RT-PCR and flow cytometry (Supplementary Figures 3 and 4). We also identified a significant correlation between CD27 gene expression and protein expression (*P*<0.001) using flow cytometry and Affymetrix data collected at diagnosis for 37 patients (Supplementary Figure 5).

**Figure 3:**
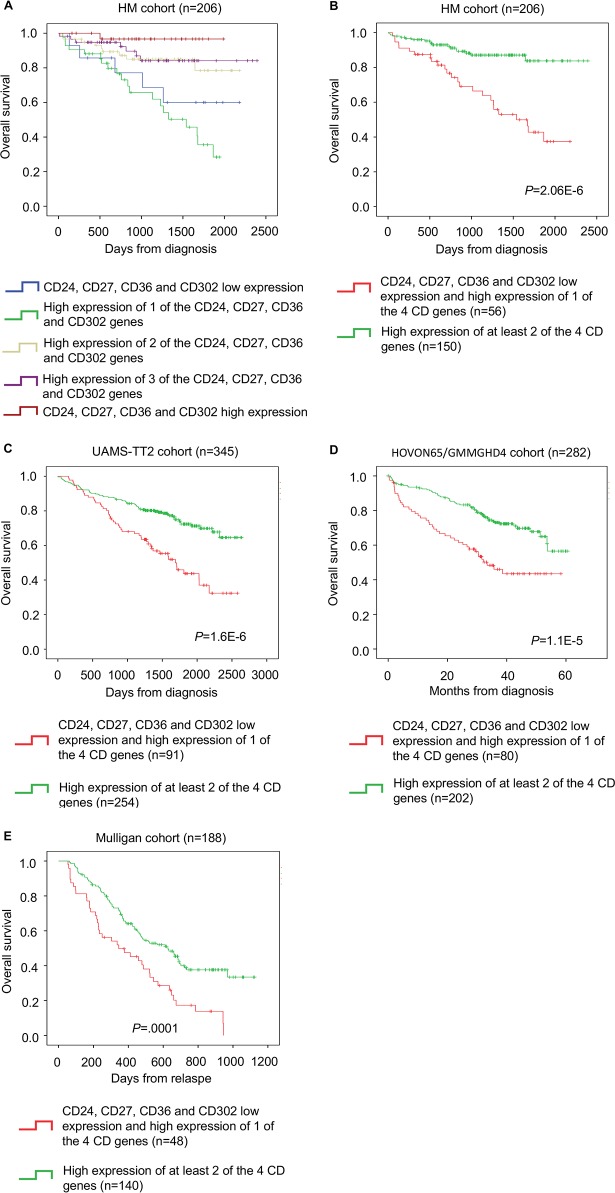
The CD gene risk score predicts overall survival in patients with MM **(A)** Kaplan-Meier estimates of the overall survival in patients from the HM cohort with high CD24, CD27, CD36 and CD302 expression (red) or low expression of one (purple), two (yellow), three (green) or all four genes (blue). When two consecutive groups showed no significant difference, they were merged. **(B)** This process led to the identification of two groups in the HM cohort: low-risk (high expression of at least two of the four genes; green) and high-risk patients (low expression of all four genes or high expression of only one of the four genes; red). The CD gene risk score prognostic value at diagnosis was confirmed in two other independent cohorts, **(C)** TT2 cohort and **(D)** HOVON65/GMMGHD4 cohort, as well as **(E)** in the Mulligan cohort (n=188).

These four CD genes were then used to create a CD gene risk score. To combine the prognostic information of *CD24, CD27, CD36* and *CD302* expression, first a simple staging scoring was built by splitting patients from the HM cohort in five groups according to *CD24, CD27, CD36* and *CD302* expression in their MMC samples (group 1: low expression of all four genes; group 2: high expression of only one; group 3: high expression of two; group 4: high expression of three of the four genes; group 5: high expression of all four genes). Kaplan Meier analysis of the five different groups (Figure 3A) was performed and when two consecutive groups did not show any significant difference, they were merged. This approach resulted in two groups with different OS. Group 1 (72.8% of all patients) comprised patients with high expression of all or two or three of the four CD genes, whereas group 2 (the remaining 27.2%) included patients with high expression of only one CD gene or low expression of all CD genes (Figure 3B). Group 1 (low risk) had a not reached median OS and group 2 (high risk) had a median OS of 50.6 months (*P* = 2.06E-6) (Figure 3B).

The prognostic value of the CD gene risk score was validated in two independent cohorts of newly diagnosed patients (TT2 cohort; n=345 patients and HOVON65/GMMGHD4 cohort; n=282 patients). Again, in both cohorts, in group 1 (low risk) the median OS was not reached, whereas in group 2 (high risk) the median OS was 55.5 months (*P* = 1.6E-6) in the TT2 cohort and 33.4 months (*P* = 1.1E-5) in the HOVON65/GMMGHD4 cohort (Figure 3C and 3D).

**Figure 4:**
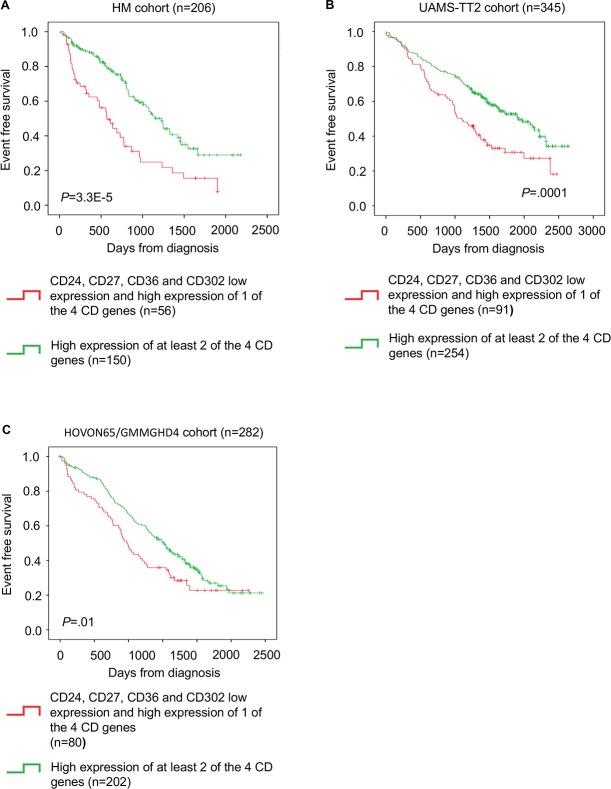
CD gene risk score predicts event-free survival in patients with MM **(A)** Kaplan-Meier estimates of event-free survival in patients with high expression of at least two of the four genes (green; low-risk group) or low expression all four genes or high expression of only one (red; high-risk group) in the HM cohort **(B)** TT2 cohort and **(C)** HOVON65/GMMGHD4 cohort.

The CD gene risk score could also predict the event-free survival (EFS) in the HM cohort (*P* = 3.3E-5) (Figure 4A). This prognostic value was validated in the TT2 (*P* =.0001) and HOVON65/GMMGHD4 cohorts (*P* =.01) (Figure 4B and 4C).

We then investigated whether the CD gene risk-score could have a prognostic value for patients at relapse using a cohort of relapsing patients treated with bortezomib (Mulligan cohort: n=188). The CD gene risk score could separate patients in two groups with significantly different OS (median OS of 11.3 months for the high-risk group and of 23.8 months for the low-risk group; *P* = 0.0001) (Figure 3E).

### COX univariate and multivariate analyses of the CD gene risk score

**Figure 5:**
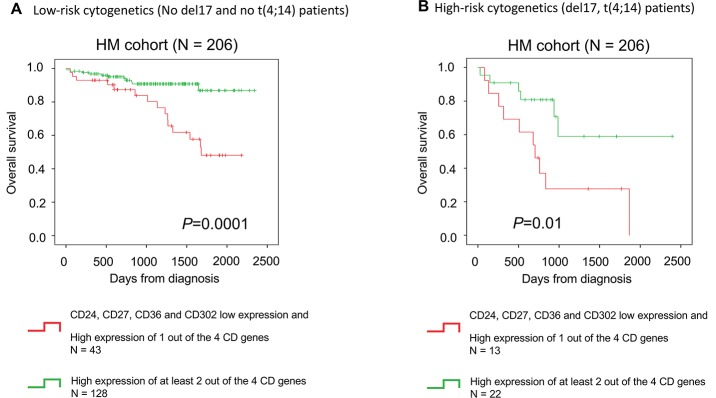
CD gene risk score is a prognostic factor in low-risk and high-risk patients based on cytogenetics The CD gene risk score predicts overall survival in **(A)** patients with MM independently of cytogenetics. The CD gene risk score has a **(B)** prognostic value in low-risk (No del17 and no t(4;14) patients) and high-risk patients based on cytogenetics (del17 and t(4;14) abnormalities).

The CD gene risk score prognostic value was then compared with that of conventional prognostic factors and other gene expression-based risk scores. In univariate COX analyses, the CD gene risk score, International Staging system (ISS) [26], β2-microglobulin (β2M) and albumin levels, t(4;14) translocation, deletion of 17p (del17p), high-risk score (HRS) [27], Intergroupe Francophone du Myelome (IFM) score [28], gene expression-based proliferation index (GPI) [29] and risk stratification (RS) score [30] showed a prognostic value in both the HM and TT2 cohorts (Table 3). In 2 by 2 analyses with ISS, β2M, t(4; 14), HRS, GPI and RS, the CD gene risk score was still independently associated with OS in the HM cohort (Table 3). In the TT2 cohort, it remained significant when tested with t(4;14), HRS, IFM score, GPI and RS score (Table 3). When all parameters were tested together, the prognostic value of the CD gene risk score, β2M, t(4; 14), IFM score and RS score remained significant in the HM cohort. In the TT2 cohort, the CD gene risk score, t(4; 14) and HRS remained independent prognostic factors (Table 3). Furthermore, the CD score remains a prognostic factor in low-risk and high-risk MM patients based on cytogenetics (Figure 5).

**Table 3:** Cox univariate and multivariate analyses to model overall survival (OS) in the HM (n=206 patients with MM) and TT2 (n=345 patients with MM) cohorts relative to the CD gene risk score, conventional prognostic factors and other gene expression-based risk scores

Univariate COX analysis	HM-cohort		TT2-cohort	
Prognostic variable	Proportional hazard ratio	*P*-value	Proportional hazard ratio	*P*-value
CD-score	0.24	<0.0001	0.41	<0.0001
ISS	1.84	0.002	NA	NA
B2m	1.1	<0.0001	NA	NA
t(4 ;14)	3.32	<0.0001	2.21	0,001
del17p	3.44	0.02	NA	NA
HRS	2.37	0.01	4.67	<0.0001
IFM score	2.49	0.01	1.78	0,004
GPI	2.54	<0.0001	1.75	<0.0001
RS	4.16	<0.0001	1.91	<0.0001
**2 by 2 Multivariate COX analysis**	**HM-cohort**		**TT2-cohort**	
**Prognostic variable**	**Proportional hazard ratio**	***P*-value**	**Proportional hazard ratio**	***P*-value**
CD-score	0.27	<0.0001	NA	NA
ISS	1.63	0.01	NA	NA
CD-score	0.23	<0.0001	NA	NA
B2m	1.1	<0.0001	NA	NA
CD-score	0.27	<0.0001	0.41	<0.0001
t(4 ;14)	2.59	0,007	2.2	<0.0001
CD-score	0.23	<0.0001	NA	NA
del17p	2.8	0.05	NA	NA
CD-score	0.23	<0.0001	0.53	0.002
HRS	2.54	0.009	3.69	<0.0001
CD-score	0.26	<0.0001	0.43	<0.0001
IFM score	1.83	NS	1.58	0.02
CD-score	0.28	<0.0001	0.46	<0.0001
GPI	2.22	0.003	1.46	0.01
CD-score	0.32	0,001	0.47	<0.0001
RS	3.76	<0.0001	1.65	0.001
**Multivariate COX analysis**	**HM-cohort**		**TT2-cohort**	
**Prognostic variable**	**Proportional hazard ratio**	***P*-value**	**Proportional hazard ratio**	***P*-value**
CD-score	0.24	<0.0001	0.57	0.009
ISS	0.92	NS	NA	NA
B2m	1.1	0.001	NA	NA
t(4 ;14)	2.91	0.02	2.13	0.003
del17p	1.26	NS	NA	NA
**Multivariate COX analysis**	**HM-cohort**		**TT2-cohort**	
**Prognostic variable**	**Proportional hazard ratio**	***P*-value**	**Proportional hazard ratio**	***P*-value**
HRS	1.52	NS	2.74	<0.0001
IFM score	0.24	0.02	0.95	NS
GPI	1.38	NS	1.14	NS
RS	3.15	0.005	0.95	NS

CD-score: CD gene risk score, ISS: International Staging system, B2M: β2-microglobulin level, t(4;14): t(4;14) translocation, del17p: deletion of 17p, HRS: high-risk score, IFM score: Intergroupe Francophone du Myelome (IFM) score, GPI: gene expression-based proliferation index, RS: risk stratification score.

### MMC from low-risk patients according to the CD gene risk score have a mature BMPC gene signature, whereas MMC from high-risk patients are characterized by high proliferation and MYC target gene-related signatures

Finally, the gene expression profiles of MMC samples from patients of the HM cohort classified as high- and low-risk based on their CD gene risk score (n=71 and n=135, respectively) were compared by using Gene Set Enrichment Analysis (GSEA) [31]. Genes related to mature BMPC (TARTE_PLASMA_CELL_VS_PLASMABLAST_UP, *P*=0.003) and to normal BMPC (ZHAN MULTIPLE MYELOMA DN, *P*=0.01) were significantly enriched in MMC samples from the low-risk CD gene risk score group (Supplementary Figure 6A). Conversely, MMC samples from the high-risk group were characterized by a significant enrichment of genes related to undifferentiated plasmablasts (MOREAUX MULTIPLE MYELOMA BY TACI DN, *P*<0.0001), MYC target genes (SCHLOSSER MYC TARGETS REPRESSED BY SERUM, *P*=0.01) and the myeloma CD1 molecular subgroup (ZHAN MULTIPLE MYELOMA CD1 UP, *P*=0.01) (Supplementary Figure 6B).

## DISCUSSION

In our study, we compared CD antigens expression between normal and malignant plasma cells to identify new potential markers to discriminate normal from malignant plasma cells, new potential therapeutic targets for monoclonal-based treatments and new prognostic factors.

Among the differentially expressed CD genes, *CD11a*, *CD19*, *CD27*, *CD52*, *CD79a*, *CD81* and *CD82* were previously shown to be downregulated in MMC compared to BMPC [32–35]. CD52 expression was shown to be correlated with chromosome 1q21 gain [10]. CD150 and CD86 protein expression was reported to be lower in MM cells compared to normal plasma cells [36]. CD19, CD27 and CD81 are also used to discriminate between normal and abnormal PCs in patients with MM [5]. Moreover, we found a significant downregulation of new surface antigens that could be useful to discriminate MMC from normal BPMC: CD5 protein like (CD5L), CD16, CD24, CD36, CD68, CD80, CD84, CD99 and CD163 (Supplementary Table 3). CD5L is involved in modulating leukocyte migration and the inflammatory response. CD5L in combination with transforming growth factor-beta inhibits B lymphocytes proliferation [37]. CD36 is a scavenger CD5L receptor and the cellular uptake of this protein is decreased in cells that downregulate CD36 [38]. CD16 (Fc γRIII) is a member of the Ig gene superfamily. Different groups did not observe *CD16* expression in the B lymphocyte lineage, including in BMPC [39] and MMC [40, 41]. Nevertheless, in MM, the serum level of soluble CD16 is correlated with the disease stage. Specifically, low CD16 expression level is associated with MM or rapidly progressing monoclonal gammopathy of undetermined significance (MGUS) compared with stable MGUS or control serum samples [42, 43]. CD68, a transmembrane glycoprotein, and CD163, a scavenger receptor of hemoglobin-haptoglobin complexes, are mainly expressed by macrophages, are linked to anti-inflammation responses [44] and are associated with poor MM prognosis [45]. CD99 is a cell surface glycoprotein involved in leukocyte migration, adhesion and transmembrane protein transport. The level of CD99 on B-cells decreases with the maturation stage and in advanced B-cell malignancies [46]. The CD24 glycolipid and the CD80 and CD84 glycoproteins are expressed on many B cells [47]. CD24 was reported to be not expressed by HMCL and MM cells from patients at relapse [48, 49]. We identified a significant downregulation of *CD24* in HMCL and in patients at relapse compared to newly diagnosed MM patients (Supplementary Figures 8 and 9). CD80 is involved in the regulation of the immune response by activating T-cells [50] and CD84 is a member of the signaling lymphocyte activation molecule (SLAM) family [51]. These downregulated proteins are mainly involved in cell adhesion, migration, proliferation and immune response. Downregulation of these proteins could be involved in MM development and MMC growth, survival and immune system escape. These new markers could be of interest to discriminate MMC from normal BMPC by multi-parametric flow cytometry at diagnosis and for the follow-up of patients with minimal residual disease.

Among the CD genes significantly overexpressed in MMC samples, CD1d, CD32 (Fc γRII), CD47, CD59 and CD200 play a role in MM pathophysiology. These proteins are involved in MMC proliferation, immune response, crosstalk between MMC and bone marrow microenvironment, antitumor cell regulation, drug resistance or bone resorption [5, 11, 13–15, 19, 52–55]. We also found significant overexpression of new potential MM surface antigens: CD2 binding protein 2 (CD2BP2), CD109, CD300a and CD320 (Supplementary Table 3). CD2BP2 interacts with CD2 and is involved in cytokine signaling, such as IL-2, in T-cells [56]. CD109 is an anchored protein with an important role in osteoclastogenesis, proliferation and tumorigenesis [57]. Like for CD47 [15, 53], CD109 targeting with anti-CD109 monoclonal antibodies could help reducing osteoclastogenesis in MM. CD300a is an immunoregulatory molecule from the same family as CD32 with a single V-like Ig domain and four immunoreceptor tyrosine-based inhibitory motifs (ITIMs) involved in immune response regulation [58]. Phosphotidylserines expressed by apoptotic cells are CD300a ligands and upon binding, the uptake of these cells by phagocytic cells is inhibited [59]. Targeting CD300a could be of therapeutic interest to increase NK cell-mediated MMC killing. CD320 (TCblR) is involved in cell metabolism and in cell cycle regulation. CD320 is strongly expressed by proliferating cells [60–62]. Furthermore, anti-CD320 antibodies inhibit the proliferation of the lymphoma cell line L3055 [60]. Monoclonal antibody-targeted therapies have shown promising results in MM [3, 4] and our study provides new potential therapeutic targets.

We then developed a CD gene risk score based on the expression level of *CD24*, *CD27*, *CD36* and *CD302* in MMC that shows a prognostic value in four independent cohorts of patients with MM. MMC from patients in the low-risk CD gene risk score group have a significant enrichment of genes related to normal and mature BMPC. Conversely, MMC from the high-risk CD-score patient group overexpress genes associated with undifferentiated plasmablasts. Accordingly, *CD24*, *CD27*, *CD36* and *CD302* are significantly overexpressed in mature BMPC compared with plasmablasts (Supplementary Figure 7). MMC samples from high-risk CD-score patients are also significantly enriched in genes related to *MYC* target genes and the myeloma CD1 molecular subgroup. *MYC* is an oncogene that acts as a transcription factor and is involved in various pathways, including cell cycle progression, apoptosis and cell transformation [62, 63]. In myeloma, *MYC* activation may be mediated through copy number changes or translocations where B-cell super-enhancers are juxtaposed to *MYC*, thus resulting in its overexpression [64]. *MYC* upregulation is associated with disease progression, evolution from MGUS to MM and poor prognosis [64]. *MYC* is involved in MM progression to the symptomatic stage and *MYC* abnormalities are detected in 54% of newly diagnosed patients [65]. The validation of this CD gene risk score at the protein level using flow cytometry could lead to a simpler, faster and cheaper prognostic tool for routine MM diagnosis and minimal residual disease follow-up. *CD24*, *CD27*, *CD36* and *CD302* gene expression could also be combined with a flow cytometry approach using the PrimeFlow^®^ RNA Assay to develop a powerful tool for risk stratification and outcome prediction in MM. Furthermore, it will be important to understand the functional role of CD24, CD27, CD36 and CD302 in MM pathogenesis and pathophysiology.

In conclusion, the CD gene risk score allows the identification of high-risk patients with MM based on the expression of four genes that encode CD surface antigens and could represent a powerful tool for simple outcome prediction in MM.

## MATERIALS AND METHODS

### Clinical samples and gene expression data

Affymetrix data from two independent cohorts of previously untreated patients with MM were used. The training cohort consisted of 206 patients with MM and was called Heidelberg-Montpellier (HM) cohort. This cohort also included five BMPC samples from healthy donors. Samples were obtained after signature of a written informed consent form in accordance with the Declaration of Helsinki and after approval by the Ethics Committees of Montpellier (DC-2008-417) and Heidelberg. After Ficoll-density gradient centrifugation, plasma cells were purified using anti-CD138 MACS microbeads (Miltenyi Biotech, Bergisch Gladbach, Germany) as previously described [66]. These data are publicly available through the ArrayExpress database (E-MTAB-372). The validation cohort (TT2 cohort) included 345 patients with MM from the University of Arkansas for Medical Sciences (UAMS, Little Rock, AR, USA). These data can be accessed at the online Gene Expression Omnibus (GSE2658). The clinical characteristics of the two cohorts have been previously described [29, 67]. Gene expression profile data from a cohort of 282 patients with MM included in the Dutch-Belgian/German HOVON-65/GMMG-HAD trial were also used (GSE19784) (HOVON65/GMMGHD4 cohort) [68]. The clinical characteristics of the this cohort have been previously described [68]. We also used Affymetrix data of 188 relapsed patients with MM subsequently treated with bortezomib (GSE9782) from the study by Mulligan *et* al. (Mulligan cohort) [69]. The XG1, XG2, XG3, XG4, XG5, XG6, XG7, XG10, XG11, XG12, XG13, XG14, XG16, XG19, XG20 and XG21 HMCLs were obtained as previously described [70]. The AMO-1, LP1, L363, U266, OPM2, and SKMM2 HMCLs were purchased from DSMZ (Braunsweig, Germany) and the RPMI8226 cell line from ATTC (Rockville, MD, USA). HMCLs were authenticated by short tandem repeat profiling and gene expression profiling using Affymetrix U133 plus 2.0 microarrays; data were deposited in the ArrayExpress public database under accession numbers E-TABM-937 and E-TABM-1088 [70].

### Gene expression profiling and statistical analyses

Gene expression data were normalized using the MAS5 algorithm and analyzed with GenomicScape (http://www.genomicscape.com) [71]. Gene expression profiles were compared using SAM. Probe sets were selected for prognostic significance using the Maxstat R function and the Benjamini Hochberg multiple testing correction. The statistical significance of differences in OS and EFS between patients’ groups was calculated by using the log-rank test. Multivariate analysis was performed using the Cox proportional hazards model. Survival curves were plotted using the Kaplan-Meier method. All analyses were done with R.2.10.1 and Bioconductor version 2.5.

### Gene set enrichment analysis

The gene expression levels of high risk and low risk MM patients according to the CD gene risk score were compared and genes with significantly different expression were identified for GSEA. GSEA was carried out by computing the overlap with canonical pathways and gene ontology annotations obtained from the Broad Institute, Cambridge, USA.

## SUPPLEMENTARY MATERIALS FIGURES AND TABLES








