# Supplementary material for: CD24, CD27, CD36 and CD302 gene expression for outcome prediction in patients with multiple myeloma
Source: Oncotarget. 2017 Oct 30;8(58):98931–44. doi: 10.18632/oncotarget.22131 (PMC5716778; doi:10.18632/oncotarget.22131)
Supplement: Supplementary file 1 [file oncotarget-08-98931-s001.pdf]

CD24, *CD27*, *CD36* and *CD302* gene expression for outcome prediction in patients with multiple myeloma

SUPPLEMENTARY MATERIALS

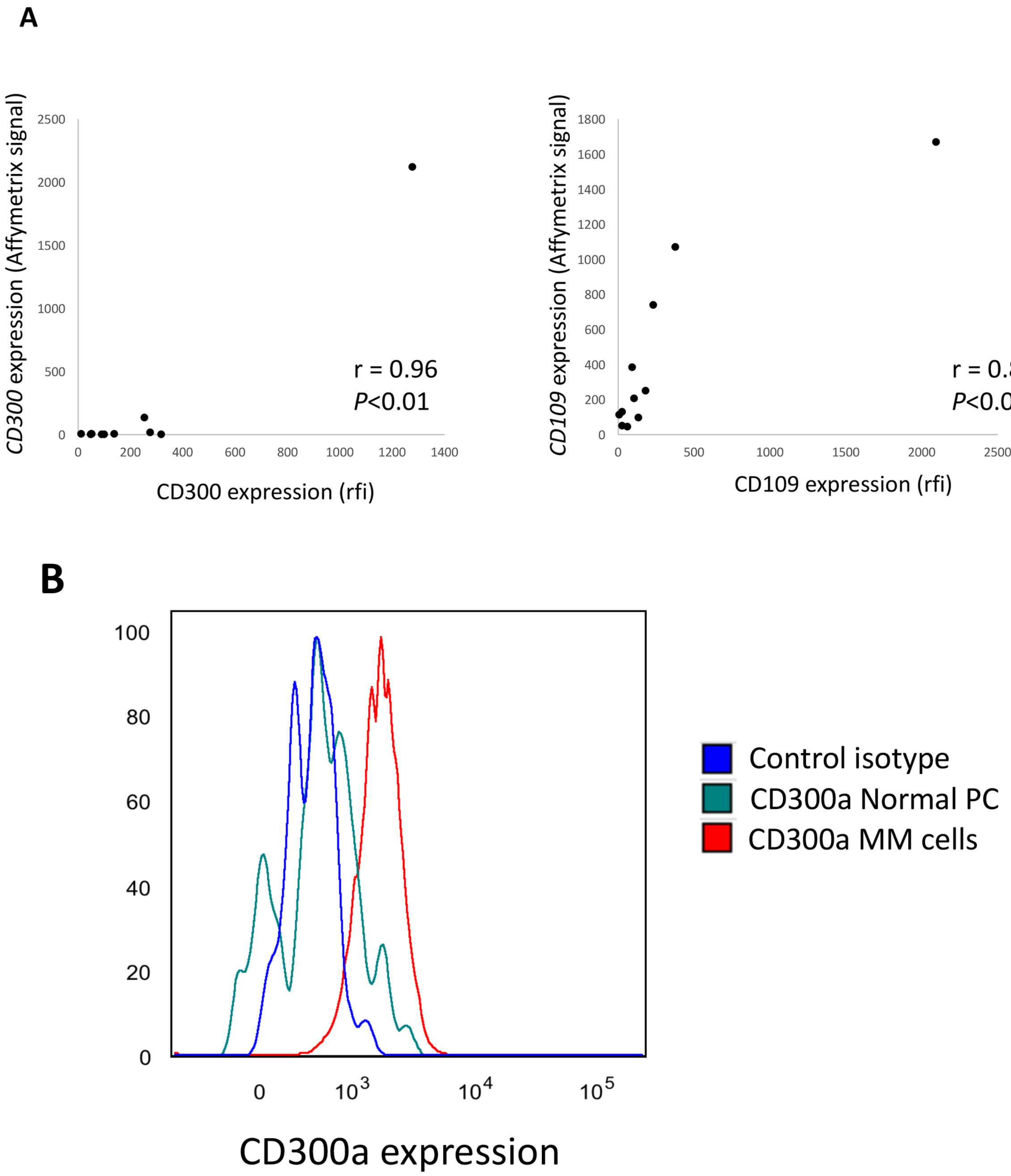

Supplementary Figure 1: Validation of Affymetrix data. **(A)** CD300a and CD109 expression in 11 HMCL were assayed by flow cytometry. The correlation between Affymetrix and relative fluorescence intensity values was determined with a Spearman's test. The coefficient correlation and *p* value are provided in the panels. **(B)** CD300a expression was analyzed by flow cytometry in normal and malignant plasma cells from a newly diagnosed MM patient.

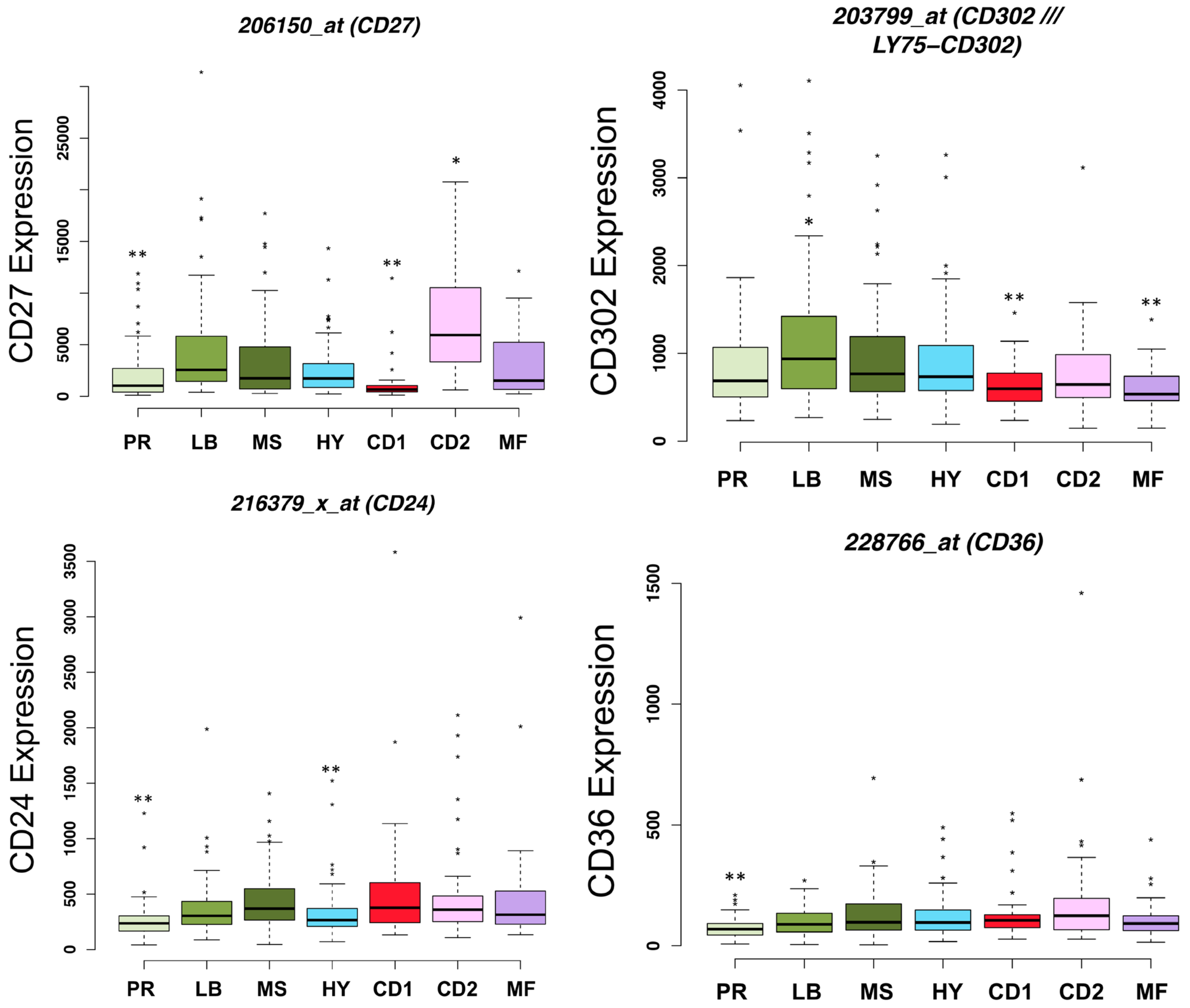

Supplementary Figure 2: CD24, CD27, CD36 and CD302 expression in MM molecular subgroups. Gene expression profiling from MMC of the patients included in the TT2 cohort were used. PR: proliferation, LB: low bone disease, MS: MMSET, HY: hyperdiploid, CD1: cyclin D1-cyclin D3, CD2: cyclin D1-cyclin D3, MF: MAF. * Indicate that CD24, CD27, CD36 or CD302 expression is significantly higher in the group compared to all the patients of the cohort (P< 0.05). ** Indicate that CD24, CD27, CD36 or CD302 expression is significantly lower in the group compared to all the patients of the cohort (*P*< 0.05) (Student t test).

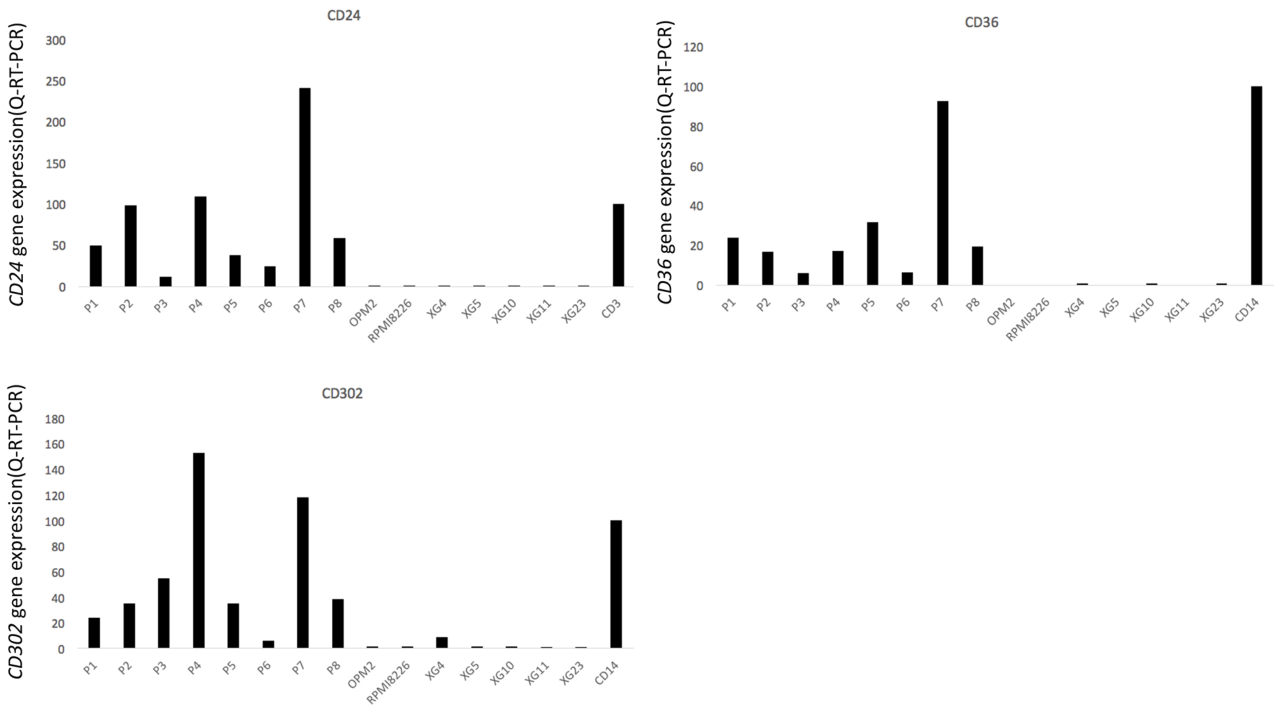

Supplementary Figure 3: *CD24, CD36* and *CD302* expression in primary MMC from patients and HMCL. *CD24, CD36* and *CD302* expression was analyzed by real-time RT-PCR in primary MMC from 8 patients and 7 HMCL. Purified CD3 cells were used as positive control for CD24.

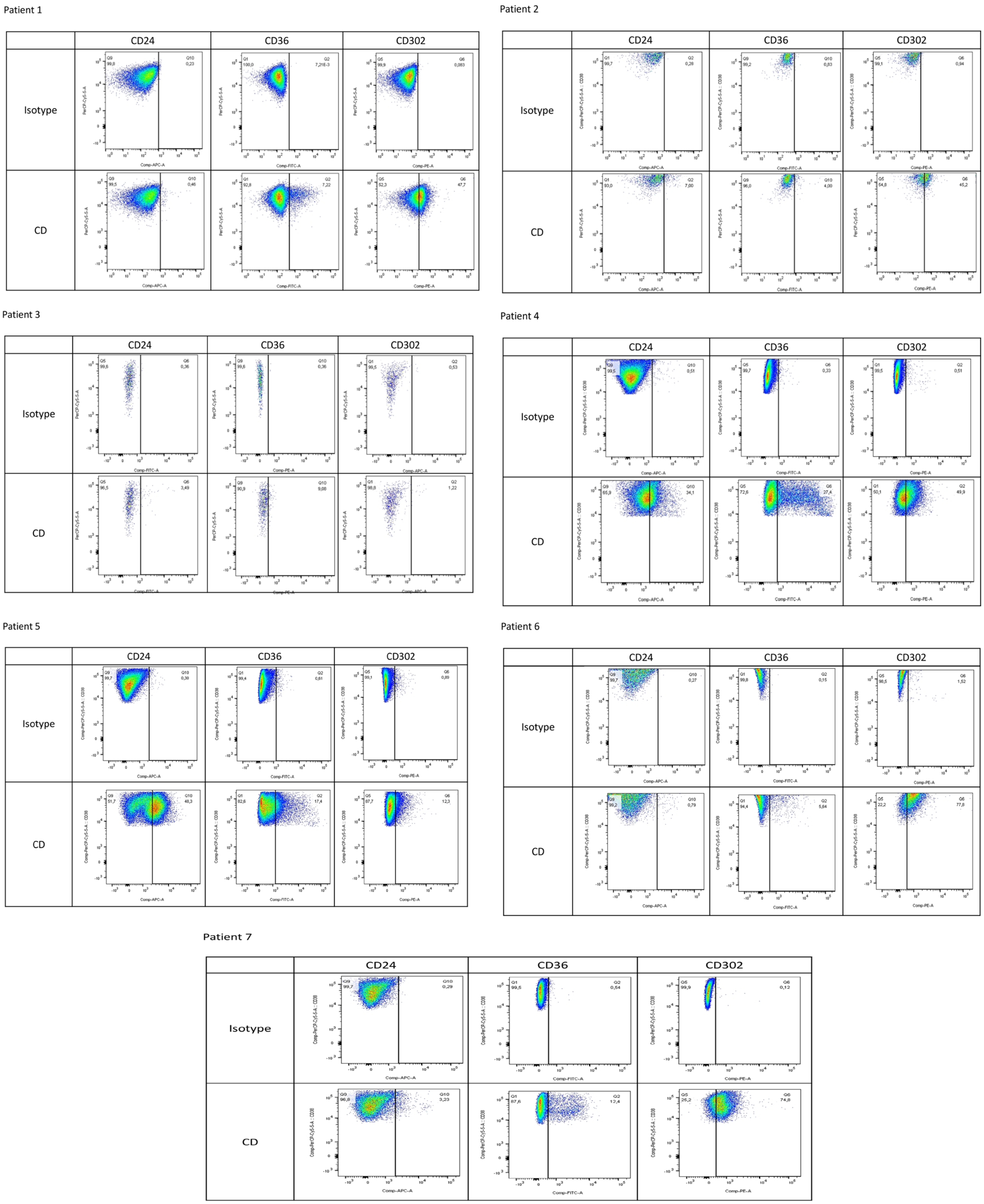

Supplementary Figure 4: CD24, CD36 and CD302 protein expression in primary MM cells from patients. Seven bone marrow samples from newly diagnosed patients with MM were analyzed using flow cytometry. Total leukocytes population was selected on FCS/SSC plot. PC were identified with CD45/CD38/CD138 antibodies. Fluorescence of CD24, CD36 and CD302 marker were compared to the corresponding isotype.

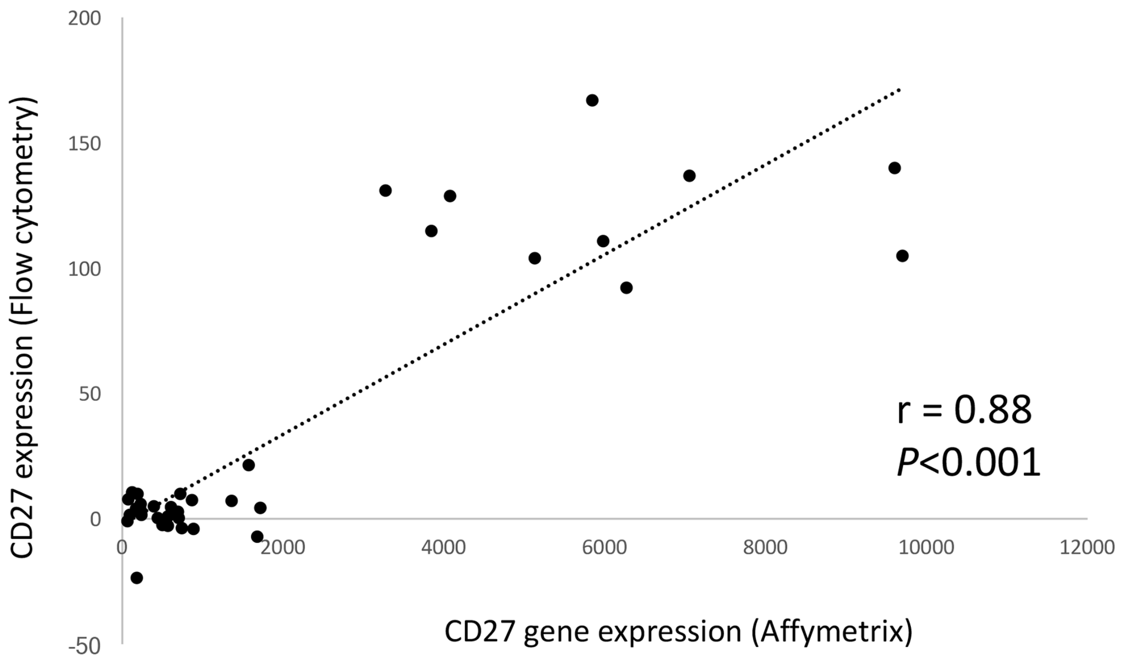

Supplementary Figure 5: Correlation between CD27 protein expression and *CD27* gene expression in MM cells. The correlation between CD27 protein expression and *CD27* gene expression was assessed in MM cells from 37 patients using Affymetrix microarrays and flow cytometry.

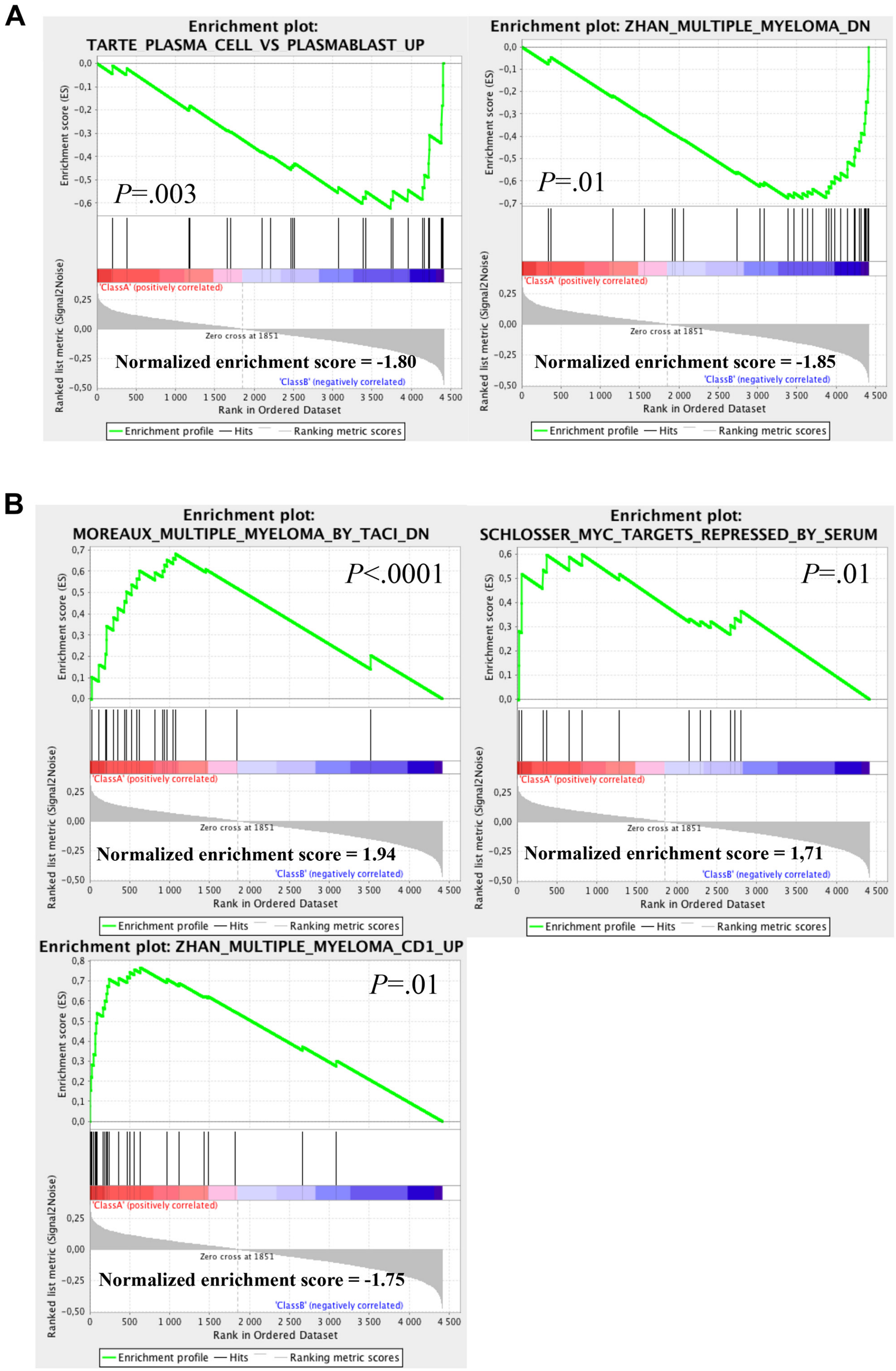

Supplementary Figure 6: Top genes that are significantly associated with **(A)** low CD gene risk score and **(B)** high CD gene risk score values in patients with MM. GSEA enrichment plot with the absolute enrichment p value and the normalized enrichment score for each gene set.

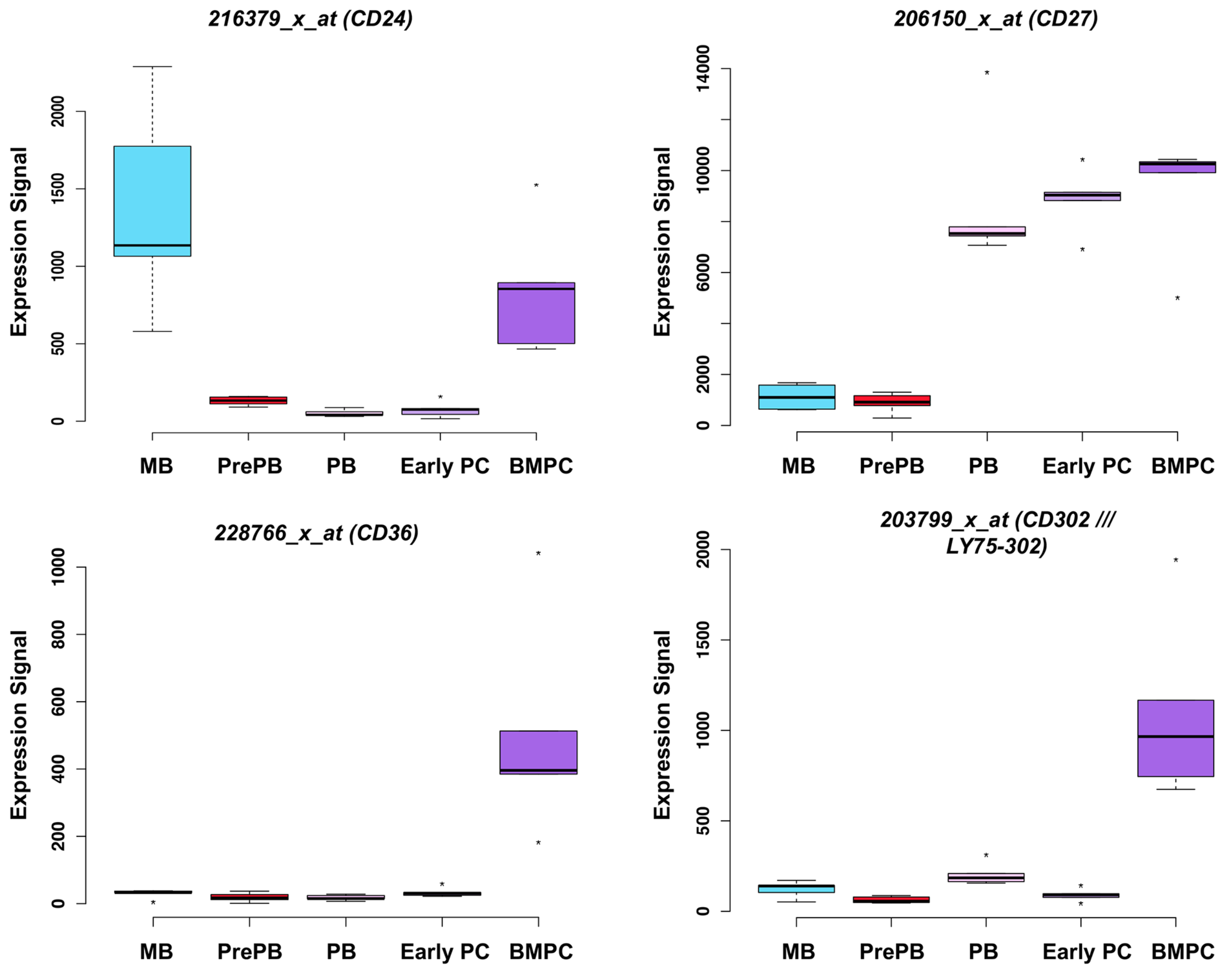

Supplementary Figure 7: *CD24, CD27, CD36* and *CD302* expression during normal plasma cell differentiation. MB: memory B cells (n=5), PrePB: preplasmablasts (n=5), PB: plasmablasts (n=5), early PC: early plasma cells (n=5), BMPC: bone marrow plasma cells (n=5).

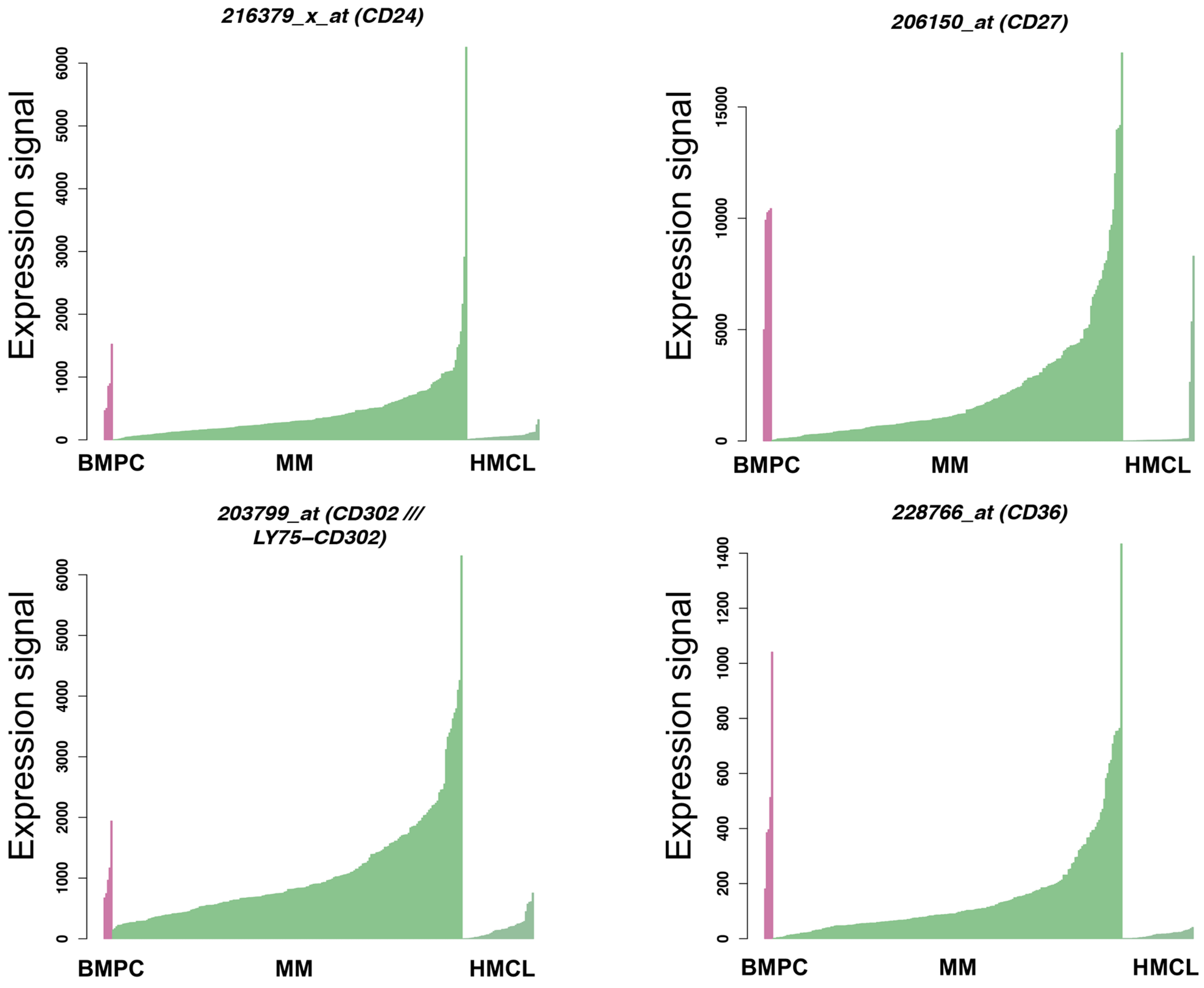

Supplementary Figure 8: ***CD24, CD27, CD36*** **and** ***CD302*** **expression in normal BM plasma cell (n = 5), MM cells from newly diagnosed patients (n = 206) and HMCL (n = 42).**

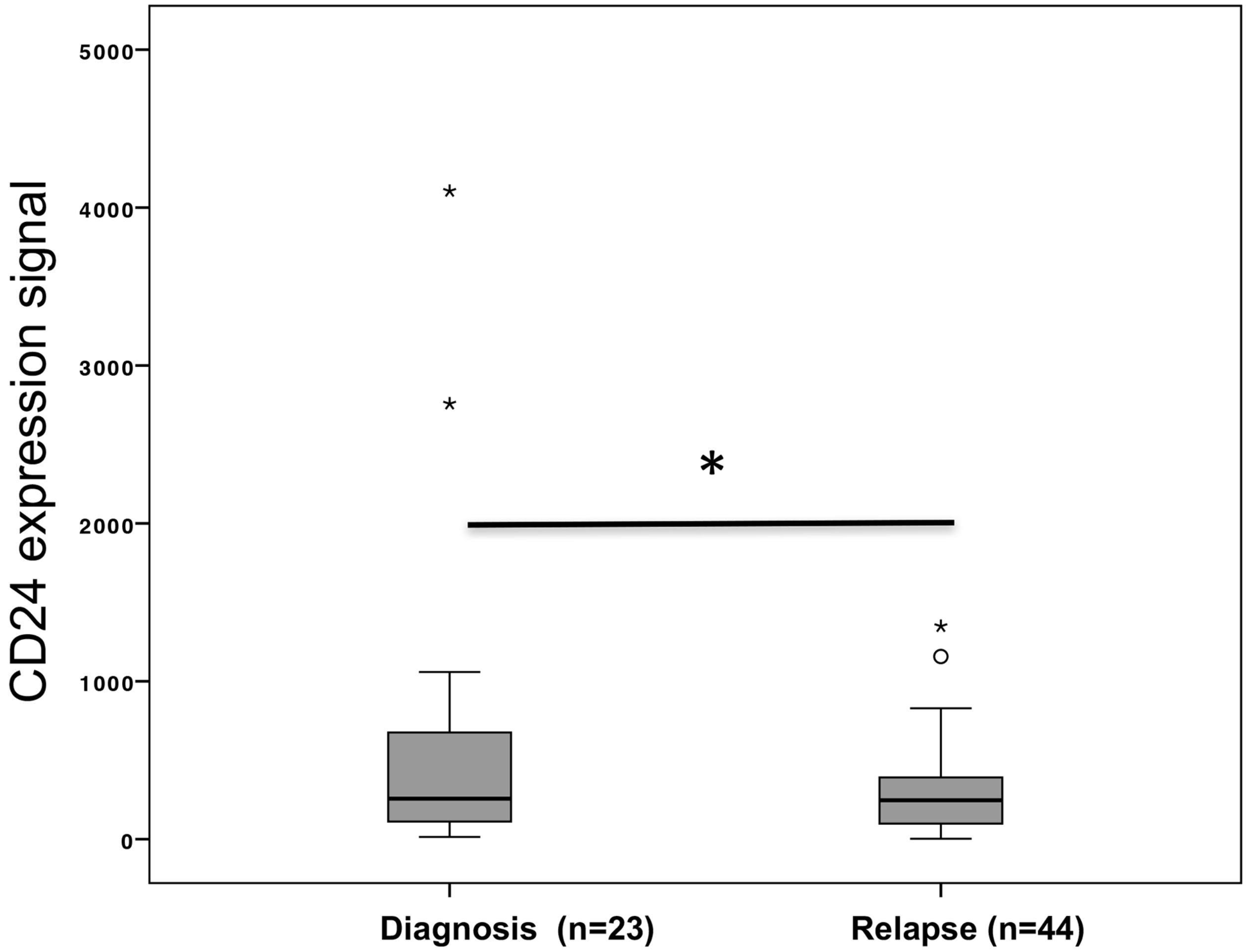

Supplementary Figure 9: *CD24* expression during normal plasma cell differentiation. CD24 gene expression using Affymetrix microarrays in purified MM cells from 23 newly diagnosed patients and 44 patients at relapse. * Indicate that *CD24* expression is significantly lower in relapsing patients compared newly diagnosed patients ($P< 0.05$) (Student t test).

Supplementary Table 1: List of the 266 probesets representative of CD molecules defined using the Human Cell Differentiation Molecules database (http://www.hcdm.org).

See Supplementary File 1

Supplementary Table 2: Cluster differentiation genes that are deregulated in HMCL compared with MMC. Gene expression was profiled in MMC samples from the HM cohort (n=206 MM patients) and HMLC samples (n=25) using Affymetrix U133 plus 2.0 microarrays. Genes that are **(A)** overexpressed and **(B)** downregulated in HMLC compared with MMC were identified using SAM supervised unpaired analysis with a 5% false discovery rate.

See Supplementary File 2

Supplementary Table 3: Description of new CD antigens deregulated in MMC compared with BMPC. We identify **(A)** new potential therapeutic targets for monoclonal-based treatments and **(B)** new potential markers to discriminate normal from malignant plasma cells.

See Supplementary File 3
